# Unventilated Indoor Coal-Fired Stoves in Guizhou Province, China: Cellular and Genetic Damage in Villagers Exposed to Arsenic in Food and Air

**DOI:** 10.1289/ehp.9272

**Published:** 2007-01-09

**Authors:** Aihua Zhang, Hong Feng, Guanghong Yang, Xueli Pan, Xianyao Jiang, Xiaoxin Huang, Xuexin Dong, Daping Yang, Yaxiong Xie, Luo Peng, Li Jun, Changjun Hu, Li Jian, Xilan Wang

**Affiliations:** 1 Department of Toxicology, School of Public Health, Guiyang Medical University, Guizhou, People’s Republic of China; 2 The 44^th^ Hospital of People’s Liberation Army, Guizhou, People’s Republic of China

**Keywords:** arsenic, coal, genetic damage, toxicity

## Abstract

**Background:**

Inorganic arsenic (iAs) is a well-known human carcinogen recognized by the World Health Organization and the International Agency for Research on Cancer. Currently, most iAs studies in populations are concerned with drinking water and occupational arsenicosis. In Guizhou province, arsenicosis caused by the burning of coal in unventilated indoor stoves is an unusual type of exposure. Because the poisoning mechanism involved in arsenicosis is as yet unknown and no effective therapy exists, progress has been slow on the prevention and therapy of arsenicosis.

**Objectives:**

We examined the relationship between arsenic (As) exposure from the burning of coal in unventilated indoor stoves and genetic damage in humans, using cellular and molecular indices. We selected villagers from Jiaole township, Guizhou province, China, who had been exposed to milligram levels of As daily via food and air contaminated by the burning of As-containing coal in unventilated indoor stoves.

**Results:**

The As-exposed subjects from Jiaole were divided into four groups according to skin lesion symptoms: nonpatients, mild, intermediate, and severe arsenicosis. Another 53 villagers from a town 12 km from Jiaole were recruited as the external control group. In the four groups of exposed subjects, As concentrations in urine and hair were 76–145 μg/L and 5.4–7.9 μg/g, respectively. These values were higher than those in the external control group, which had As concentrations of 46 μg/L for urine and 1.6 μg/g for hair. We measured sister chromatid exchange and chromosomal aberrations to determine human chromosome damage, and for DNA damage, we measured DNA single-strand breaks and DNA–protein cross-links. All measurements were higher in the four exposed groups compared with the external control group. DNA repair was impaired by As exposure, as indicated by the mRNA of O-6-methylguanine-DNA methyltransferase (MGMT), X-ray repair complementing defective repair in Chinese hamster cells 1 (XRCC1), and, to a lesser extent, by the mismatch repair gene hMSH2 mRNA. The expression of mutant-type *p53* increased with aggravation of arsenicosis symptoms, whereas the expression of p16-INK4(p16) decreased. *p53* mutated at a frequency of 30–17% in the carcinoma (*n* = 10) and precarcinoma (*n* = 12) groups. No mutation was found in *p16,* although deletion was evident. Deletion rates were 8.7% (*n* = 23) and 38.9% (*n* = 18) in noncarcinoma and carcinoma groups, respectively.

**Conclusions:**

The results showed that long-term As exposure may be associated with damage of chromosomes and DNA, gene mutations, gene deletions, and alterations of DNA synthesis and repair ability.

Although inorganic arsenic (iAs) is a well-known human carcinogen recognized by the World Health Organization (2001) and the International Agency for Research on Cancer (1987), the mechanism of carcinogenicity are not clear. Because of significant differences in arsenic (As) metabolism between experimental animals and humans, the use of animal models to evaluate the carcinogenic effects iAs has not been established successfully (Goering et al. 1999). For decades, As has been considered a nongenotoxic carcinogen because it is only weakly active or, more often, completely inactive in bacterial and mammalian cell mutation assays (Hei and Filipic 2004). Recently, increasing evidence has shown that As is a strong, dose-dependent gene and chromosomal mutagen that is capable of inducing mostly multilocus deletions (Hei et al. 1998). Experiments in mammalian cells have also shown that this induction was significantly reduced in the presence of antioxidant enzymes (Liu et al. 2002).

As early as 1976, villagers from Guizhou province in southwestern China were reported to be suffering severe symptoms of arsenicosis (Zhou et al. 1993), which was attributed to exposure to high levels of As in food, especially in corn and chili peppers, and to a lesser extent by breathing As-laden air (Finkelman et al. 2003). Villagers mined local low-grade As-containing coal from abundant, small local coal pits, with As-coal concentrations mostly in the range of hundreds of milligrams per kilogram (Finkelman et al. 1999; Zheng et al. 1999). Corn and chili peppers were dried over unventilated indoor stoves used for every day cooking and for heating during the winter months. Repeated surveys of As-coal and medical examinations have identified nine towns in four counties in southwest Guizhou province as having high levels of As in food and air (Zhang et al. 2000a). Approximately 200,000 people within the four counties were at risk of exposure to high levels of As; 3,000 cases of arsenicosis were diagnosed in the late 1990s, with approximately 2,000 of these cases in Xinren county alone (Liu et al. 2002). Over the years, various measures adopted by local governments, such as shutting down the coal pits containing high levels of As and installation of ventilated stoves, have been only minimally effective until a health education campaign was implemented in 2005 (An et al. 2005).

New cases of arsenicosis have been identified each year since 1990, although there was evidence of reduction in exposure as early as 1998. Between 1998 and 2004, collected coal samples had As concentrations of 92–816 mg/kg (Huang et al. 2002). The average concentration in indoor air during that time was 0.087 ± 0.045 mg/m^3^ (*n* = 22), which is lower than that in indoor air in 1991 [0.46 ± 0.30 mg/m^3^ (*n* = 18); Zhang et al. 2000a; Zhou et al.1993]. Urinary As concentrations declined from 130.6 ± 121.2 μg/L (*n* = 167) in 1998 to 97.0 ± 76.1 μg/L (*n* = 43) in 2004. This finding coincided with the decrease of As concentrations in indoor air. In the exposed population, skin lesions were common. Other damage included lung dysfunction, neuropathy, and nephrotoxicity. The prevalence of hepatomegaly was 20%. Approximately 200 people have died from the effects of the most severe As poisoning, which included liver cirrhosis, ascites, and liver and skin cancers. Treatment of individuals who have arsenicosis was difficult because of a long exposure time of more than 30 years (Zhou et al.1993) and the high level of As exposure in the population.

In this article, we report the results of a series of investigations between 1998 and 2004, which included a large number of individuals with arsenicosis. To look for cellular and molecular biomarkers of exposure, we collected blood and skin samples from villagers who had been exposed to As. We analyzed the effects of As exposure on chromosome and DNA damage, DNA synthesis and repair, and tumor suppressor gene mutations. One of our goals was to identify molecular biomarkers for early diagnosis that may be applicable to populations exposed to much lower levels of As, usually found in drinking water.

## Materials and Methods

### Subjects

We consulted a database maintained by the Guizhou Provincial Office of Endemic Disease to identify populations exposed to As (Yang et al. 1998). All subjects in our study resided in Jiaole township in Xinren county and gave informed consent. Our team examined the target population in 1998, with a total of 184 villagers who had been exposed to As agreeing to participate in our study. Arsenicosis symptoms were categorized based on the degree of symptoms: nonpatient (*n* = 19), mild (*n* = 49), intermediate (*n* = 54), and severe (*n* = 62). Symptoms were classified according to the Chinese National Arsenicosis Diagnosis Standard protocol (Yu et al. 2007). Two control groups were included in this study. First, non-patients (*n* = 19), defined as individuals showing no symptoms of arsenicosis, from Jiaole, were designated the internal control group. The second, designated the external control group, comprised 53 villagers from Ma Jiatun township (approximately 12 km from Jiaole) who did not use coal containing high levels of As (Table 1).

### Biological samples

Blood and skin samples were collected each year from 1998 to 2001 from villagers burning As-containing coal in unventilated indoor stoves. Biosamples were collected from the same villagers at each collection time; however, we could not always collect samples from all subjects at each collection time, which resulted in missing data.

In 1998 we obtained 193 blood samples (external, *n* = 25; internal, *n* = 13; mild, *n* = 44; intermediate, *n* = 49; severe, *n* = 62) in which to study cellular chromosome damage (Table 1). In the same year, skin samples were collected from 70 subjects who were treated surgically to alleviate pain related to arsenicosis. These samples were used to measure of *p53* and p16-INK4 (*p16*) gene expression (Table 2). In 1999 we obtained a second batch of blood samples from 156 patients, 19 internal controls, and 41 external controls in which to measure DNA damage (Table 1). In 2001 a third batch of blood samples was collected from 70 subjects for analysis of *p53* and *p16* mutations (Table 2). Also, in 2001, a second batch of skin samples from 61 subjects who had undergone surgery was obtained for DNA gene repair study (Table 3). On the basis of dermal pathology, the subjects from 2001 study were categorized into four groups: group A, general pathological changes (hyperplasia) with 22 subjects belonging to mild (*n* = 8), moderate (*n* = 7), severe (*n* = 7) arsenicosis groups; group B, hyperkeratosis with 22 subject belonging to mild (*n* = 3), moderate (*n* = 7), and severe (*n* = 12) arsenicosis group; group C, precancerous lesion with 12 subjects drawn from moderate (*n* = 5) and severe (*n* = 7) arsenicosis groups; and finally, group D, cancerous lesion with 18 subjects including squamous carcinoma, basal cell carcinoma, and Bowen disease from mild (*n* = 1), moderate (*n* = 3), and severe (*n* = 14) arsenicosis groups.

Urine samples were collected in acid-washed plastic containers. Concentrated hydrochloric acid (1 mL HCl to 100 mL urine) was added to prevent bacterial growth (Concha et al. 1998). The samples were then frozen and stored at –80°C (Crecelius et al. 1986). Collected skin tissue was flash frozen in liquid nitrogen and stored at –80°C (He et al. 2004). Blood samples were kept on dry ice before being transported to our laboratory and kept frozen at –80°C.

### Biomarkers of biological response

We chose biomarkers of mutation and abnormal gene expression because they can indicate molecular changes before the occurrence of cancer and because of their potential usefulness in early diagnosis (Klaassen 2001). We used a standard protocol, and the parameters and analytical procedures are described in detail in the citations above.

### Chromosome and DNA damage

**Figure 1:**
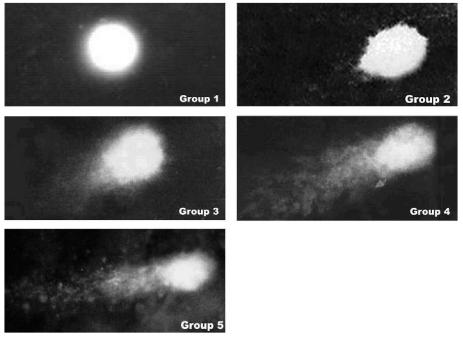
Results of DNA single-strand breaks with SCGE (400×). Group 1, external control group; group 2, internal control group; group 3, mild arsenicosis group; group 4, intermediate arsenicosis group; group 5, severe arsenicosis group (see Table 1 for urinary and hair As concentrations).

Sister chromatid exchanges (SCEs), chromosomal aberrations (CAs), and micronuclei (MN) (Table 1) in peripheral blood were blind counted (Xie et al. 1999). Spontaneous DNA synthesis (DS) and unscheduled DNA synthesis (UDS) were assayed with a liquid scintillation counter (Beckman Coulter Inc., Fullerton, CA, USA; Zhang et al. 2000c). DNA–protein cross-links (DPCs) were measured by an ^25^I-post labeling assay (Zhang et al. 2000c). DNA single-strand breaks (SSBs) were determined by single-cell gel electrophoresis (SCGE; Zhang et al. 2000b) and the yield of these breaks was measured using the DNA COMET assay (Figure 1).

### Expression of P53 and P16 proteins

It is well known that wild-type P53 protein is a nuclear phosphoprotein coded by the *p53* tumor suppressor gene. If the DNA is damaged, wild-type *p53* shuts off cell duplication and initiates a “suicide system” to kill those cells with damaged DNA. In contrast, if wild-type *p53* mutates into mutant-type *p53*, cells with damaged DNA may enter the S phase prematurely and produce CAs (Vogelstein 1990). P16 protein is a known inhibitor of cyclin-dependent kinase 4 (CDK4) that binds to CDK4 in the G_1_ phase of the cell cycle to down-regulate its activity. It inhibits the progression of cells, especially those with DNA damage from G_1_ to S phase. As a result, cell division and growth are reduced (Serrano 1993; Sherr 1993).

The immunohistochemical technique is well established for use in clinical histopathological diagnosis (Chen et al. 2005; Editorial Board of the *Chinese Journal of Pathology* 1996). The expression of P53 and P16 (Table 2) was measured by immunohistochemical methods in skin tissue samples, most of which (*n* = 70) were collected in 1998 (Hong et al. 2000; Hu et al. 2003). The mouse monoclonal P53 and P16 antibodies and EnVision+ System kit were purchased from Dako (Dako North America Inc., Carpinteria, CA, USA). In our study the quantitative data for these percentages were transformed into two qualitative types: positive or negative. For P53 and P16 protein, a subject with more than 1% positive cells was regarded as positive.

### *Mutation of* p53 *and* p16 *genes.*

**Figure 2:**
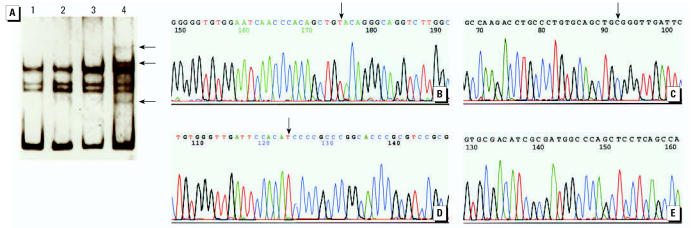
Nucleotide sequence of *p53* and *p16*. (*A*) PCR-SSCP analysis of *p53* exon 5. Arrow indicates the abnormal band. Lane 1: peripheral blood of healthy people; lanes 2–4: peripheral blood of patients with arsenicosis. (*B*) Nucleotide sequence of *p53*, codon 143 (GTG→GTA). Arrow indicates the mutation site (sample of arsenicosis patient with precarcinoma: urinary As, 106.29 μg/L; hair As, 21.64 μg/g. (*C*) Nucleotide sequence of *p53*, codon 146 (TGG→CGG). Arrow indicates the mutation site (sample of arsenicosis patient with carcinoma: urinary As, 79.14 μg/L; hair As, 16.42 μg/g). (*D*) Nucleotide sequence of *p53*, codon 151 (CCC→TCC). Arrow indicates the mutation site (sample of arsenicosis patients with carcinoma: urinary As, 274.77 μg/L; hair As, 6.18 μg/g). (*E*) Part of the noncoding strand in *p16* exon 2 (sample of arsenicosis patient with hyperkeratosis: urinary As, 111.75 μg/L; hair As, 9.94 μg/g).

Polymerase chain reaction (PCR) single-strand conformation polymorphism (SSCP), as well as PCR cloning and sequencing, were used to detect the mutation of *p53* exons 5–8 and *p16* exon 2 in 60 peripheral blood samples of subjects (Table 2; Figure 2) collected in 2001 (Pan et al. 2004;). Point mutation of *p53* occurs mainly in exons 5–8 (Hasegawa et al. 1995; Vet et al. 1996; Xu et al. 2001); The PCR primers for *p53* were as follows: first pair of primers—forward, 5′-GTGAGGGG-GCTCTACACAAG-3′; reverse, 5′-ACCA-GCGTGTCCAGGAAG-3′; second pair of primers—forward, 5′-CTTCCTGGACAC-GCTGGT-3′; reverse, 5′-GTCCTCACCT-GAGGGACCTT-3′.

Point mutation of *p16* gene occurs mainly in exon 2 (Kamb et al. 1994; Nobori et al. 1994). To obtain a suitable length of segment for nondenatured polyacrylamide gel, *p16* was amplified by two pair primers, the primer sequences are are as follows: first pair of primers—forward, 5′-GTGAGGGGGC-TCTACACAAG-3′; reverse, 5′-ACCAGC-GTGTCCAGGAAG-3′; second pair of primers—forward, 5′-CTTCCTGGACA-CGCTGGT-3′; reverse, 5′-GTCCTCAC-CTGAGGGACCTT-3′. The PCR mixture contained 1 × PCR buffer; dATP,dCTP, dGTP,dTTP (2 μM each); primers (100 nM); MgCl_2_ (1.5 mM); Taq DNA polymer (2 U); and template DNA (200 ng) in a final volume of 50 μL. The PCR program was set for an initial denaturation at 95°C for 5 min, 35 cycles of denaturation at 94°C for 50 sec, annealing at 56–65°C (depending on the type of primer pair used) for 40 sec, extension at 72°C for 50 sec, and final extension at 72°C for 5 min. PCR products were electrophoresed on 1.5% agarose gels and observed under ultraviolet illumination. Five micro-liters of PCR product was mixed with 5 μL of denatured solution, which was denatured at 95°C for 5 min and kept on ice for 5 min. Similarly, a PCR product was added to 8% nondenatured polyacrylamide gel and electrophoresed with 150 V of constant voltage for 3 hr. After electrophoresis the gel was removed and stained with silver.

PCR products were inserted into vector pMD 18-T, then transformed to the competent DH5α cell, prepared in LB medium containing AMP100. Plasmid DNA was extracted using E.Z.N.A Plasmid Miniprep Kit I (Omega Bio-Tek, Inc., Doraville, GA, USA); recombinant DNA was screened with EcoRI, HindIII, and sequenced in Takara Biotechnology Co. (Dalian, China).

### Expression of DNA repair gene

Expressions of O-6-methylguanine DNA methyltransferase (MGMT), X-ray repair complementing defective repair in Chinese hamster cells 1(XRCC1), and mismatch repair gene (hMSH2) mRNA (Table 3) were analyzed by *in situ* hybridization in 61 skin tissue samples. The samples were from arsenicosis patients who had undergone surgery in 2001 (Chen et al. 1999; Zhang et al. 2005). Tissue samples were analyzed as follows: The sections were treated with 3% hydrogen peroxide, digested in proteinase, and prehybridized at 37°C. Sections were then hybridized overnight and blocked in paraffin or mineral oil. Mouse biotin-antidigoxin antibody was applied for 60 min at 37°C, followed by treatment with streptavidin–biotin–peroxidase complex (Wuhan Boster Bioengineering Ltd., Wuhan, Hubei, China) for 20 min at 37°C, with biotin–peroxidase for 20 min at 37°C, and stained with DAB (Wuhan Boster Bio-engineering Ltd., Wuhan, Hubei, China). The sections were then refiltered, dehydrated, cleared and enveloped and visualized using a microscope. The *in situ* hybridization kit, DAB stain kit, and *in situ* hybridization special cover-slices were purchased from Wuhan Boster Biological Engineering Limited Corp. (Wuhan, China). After *in situ* hybridization, positive samples exhibited brownish yellow inclusions in the cytoplasm. The percentage of samples was calculated as follow: positive cases ÷ detected cases × 100%. Skin samples from control groups were taken from nonarsenicosis patients receiving undergoing other medical procedures at that hospital at that time.

### Statistical analyses

SPSS (version 11.0, http://www.spss.com) was used for analysis of variance (ANOVA), linear correlation and regression, and the chi-square test. Results were reported as statistically significant when *p* < 0.05.

## Results

### Arsenic exposure and chromosome and DNA damage

For the external control group, the average concentrations of urinary As and hair As were 46 μg/L and 1.6 μg/g, respectively (Table 1). Urinary As concentrations in the four exposed groups were 2–3 times that in the external control group, with *p* < 0.01. Compared with the external control group, hair As concentrations were 3– 5 times that in the exposed groups, with *p* < 0.01. It is worth noting that concentrations of urinary As (~ 76 μg/L) and hair As (~ 5.4 μg/g) in the internal control group, that is, subjects without diagnosable arsenicosis symptoms, were statistically significantly (*p* < 0.05) lower than those in the three other groups with mild, moderate, or severe arsenicosis (Table 1). In addition, both urinary and hair As concentrations increased as the degree of arsenicosis increased.

Consistent with the statistically significant difference of As exposure, SCE ratio, which is an indicator of chromosome damage, was found to be significantly different in all four exposed groups compared with the external control group (*p* < 0.05 or *p* < 0.01; Table 1). In addition, the SCE ratio of the severe arsenicosis group was higher than that of the internal control group (*p* < 0.05; Table 1). CAs were found to be significantly different in the three patients groups compared with the external control (*p* < 0.01; Table 1). For MN, only the severe arsenicosis group was significantly different from the external control group, internal control group, and the other two exposed groups (Table 1).

Unscheduled DNA synthesis increased gradually with increasing As concentrations in urine and hair (Table 1), whereas spontaneous DNA synthesis showed a dramatic decrease from approximately 1,150 cpm for the external control group to approximately 200 and 300 for all four exposed groups. DNA–protein crosslinks increased with increasing exposure (Table 1). DPCs in the internal control group was much higher than that in the external control group (*p* < 0.01; Table 1). It increased remarkably in the moderate and severe groups, compared with the two control groups and the mild arsenicosis group (*p* < 0.05 or *p* < 0.01; Table 1). DNA COMET tail length (Figure 1) increased with increasing As exposure when external control, nonpatient, mild, moderate, and severe arsenicosis groups were compared.

### Arsenic exposure and abnormal expressions of P53 and P16 proteins

In patients with arsenicosis, skin pathology was classified according to four groups. The positive rate in P53 protein expression was 89% (16/18), 73% (8/11), 33% (7/21) and 17% (3/18) for the carcinoma, precarcinoma, hyperkeratosis, and common pathological changes groups, respectively (Table 2). The high positive rates in the carcinoma and precarcinoma groups were statistically different from those in the common pathological changes and hyperkeratosis groups (*p* < 0.05 or *p* < 0.01; Table 2). The positive rates of P16 protein expression were 33% (6/18), 36% (4/11), 68% (15/22), and 79% (15/19) for the carcinoma, precarcinoma, hyperkeratosis, and common pathological changes groups, respectively. The low positive rate in the carcinoma group was statistically different from those in all other groups; there is also a significant difference between the precarcinoma group and the common pathological changes group (Table 2).

### *Arsenic exposure and* p53 *gene mutation.*

A striking difference in the *p53* mutation frequency was found between the carcinoma (30%) and precarcinoma groups (17%) compared with the hyperkeratosis (0%) and common pathological changes (0%) groups (*p* < 0.05; Table 2). Sequencing *p53* identified the following mutation types and sites: at codon 143 (GTG→GTA) in two cases, which were silent mutations; at codon 146 (TGG→CGG) in one case, resulting in a change from tryptophan to arginine in the protein; at codon 151 (CCC→TCC) in two cases, resulting in a change from proline to serine in the protein (Table 4; Figure 2). No mutation was found on *p16* exon 2.

### Arsenic exposure and abnormal expression of DNA repair genes

The positive rates of all DNA repair genes, that is, MGMT, XRCC1, and hMSH2 mRNA, increased gradually as the arsenicosis symptoms became milder (Table 3). The positive rates for MGMT mRNA were 18, 42, 69, and 73% for the carcinoma, precarcinoma, hyperkeratosis, and common pathological changes groups, respectively. The carcinoma group displayed statistically lower positive rates than those of the common pathological changes group (*p* < 0.01) and the hyperkeratosis group (*p* < 0.05) but not lower than those of the precarcinoma group. Similar trends were observed for two other DNA repair genes with respect to As exposure and symptoms. In this case the low positive rate of XRCC1 mRNA in the carcinoma group (27%) was significantly lower than that for the common pathological changes group (77%; *p* < 0.01). The low positive rate of hMSH2 mRNA (36%) for the carcinoma group was not statistically significant from the other groups.

## Discussion

Our results clearly demonstrate that in a population exposed to As primarily through consumption of food cooked on coal-burning unventilated indoor stoves and, to a much lesser extent, inhalation of the contaminated air from these stoves, cellular and genetic material exhibited changes in most cases consistent with the As exposure level and symptoms. In individuals with arsenicosis, DNA was damaged, DNA repair was impaired, and mutations or deletions of *p53* and *p16* were evident. Our findings suggest: *a*) SCE and CAs are sensitive biomarkers for human chromosome damage induced by As exposure; *b*) DNA single-strand breaks and DPCs can be used to monitor DNA damage in populations exposed to As; *c*) *p53* mutations and *p16* deletions were associated with carcinogenicity due to As exposure; and *d*) the decrease in DNA repair may be another important mechanism in arsenicosis carcinogenicity.

### Biomarkers of As-induced human chromosome damage

As-induced chromosome damage appeared to occur earlier than clinical symptoms, as shown by the higher SCE, CA, and MN values in the exposed groups compared with the external control group. However, among the three indicators of chromosome damage, the MN seems to be the least sensitive because the difference is not statistically significant between the internal control, mild, and intermediate arsenicosis groups compared with the external control (Table 1). SCE was found to be the most sensitive indicator of arsenic exposure because it was the only biomarker that displayed a statistically significant difference between the internal and the external control groups (Table 1). The CAs was less sensitive than SCE, but more sensitive than MN (Table 1). Both mammals and humans exposed to As had increased frequencies of MN and CAs, which led to SCE abnormalities (Wilson et al. 2002). Gurr et al. (1993) found that when sodium arsenite was used to treat G_2_-phase Chinese hamster ovary cells, chromosome condensation and breakage were induced, indicating MN development during interphase.

### Biomarkers of DNA damage

The present study also showed that SCGE, a sensitive and common assay, could be used to monitor DNA damage of populations exposed to As. Arsenic caused DNA single-strand breaks in As-exposed populations, with a wide range of symptoms. The breaks occurred notably earlier than clinical symptoms (Figure 1). This is consistent with DNA damage determined by SCGE in human lymphocytes cultured *in vitro* with a high concentration of arsenate (0.2–1.5 mmole/L) (Hartmann and Speit 1994). Furthermore, our results and those from cell culture studies (Li et al. 2001) showed that As induced DNA strand breaks and exhibited a dose–response relationship.

DNA–protein cross-links are thought to be important genotoxic lesions induced by environmental agents and carcinogens, such as ultraviolet light (Smith 1962), formaldehyde (Cosma et al. 1988), and *cis*- or *trans*-platinum (II) diammine dichlorides (Banjar et al. 1984). Unlike strand breaks and other DNA lesions that are readily repaired, DPCs are relatively persistent in the cells (Lei et al.1995; Oleinick et al.1987). Because of a poor repair capacity, DNA–protein complexes still exist during DNA replication, which may cause loss of important genetic material and even inactivate tumor suppressor genes (Costa 1991; Lei et al.1995). In our study, the extent of DNA–protein cross-linking in the internal control groups and the three exposed groups was signifcantly higher than that in the external control group. There exists a positive relative relationship between DPC and As poisoning (Table 1). This suggests that DNA–protein cross-linking can be used to monitor genetic damage from As exposure. The formation of DPCs may be one of the mechanisms that induces mutation and perhaps cancers in arsenicosis patients.

### Gene mutations, deletions, and As exposure

*p53* mutations were found by PCR-SSCP in the peripheral blood samples of arsenicosis patients with precarcinomas and carcinomas. The mutation frequencies, 17 and 30% for the precarcinoma and carcinoma groups, respectively, were lower than those reported previously in tumor tissues of arsenicosis cancer patients from Taiwan (Hsu et al. 1998; Shibata et al. 1994). The mutation frequency of *p53* exons 5–8 was determined by PCR-SSCP to be 62%, or in 8 of 13 bladder cancer patients exposed to As in drinking water from the Black Foot disease area in Taiwan (Shibata et al. 1994). The mutation frequencies of *p53* exons 2–11 were 28.6%–55.6% in skin cancer patients with Bowen’s disease, basal cell carcinoma, or squamous cell carcinoma, again from the Black Foot disease area in Taiwan (Hsu et al. 1998). These differences in DNA mutation frequencies may be attributed to sample type: peripheral blood versus tumor tissue. Several considerations prompted us to investigate DNA samples from peripheral blood: *a*) Arsenic is harmful to many organs and can induce to tumorigenesis. *b*) Tumorigenesis has multiple stages and often occurs over a long period. Monitoring mutations in blood samples taken over time provides a better opportunity than tissue samples for investigating gene mutations and tumorigenesis, as tissue samples are usually more difficult to obtain.

The results of cloning and sequencing showed that the mutation sites were found at codon 143 (GTG→GTA) in two cases, which was a silent mutation; at codon 146 (TGG→CGG) in one case, which resulted in a change from tryptophan to arginine in the protein; and at codon 151 (CCC→TCC) in two cases, which resulted in a change from proline to serine in the protein (Figure 2). Hsu et al. (1998) reported similar results in 16 skin cancer patients in Taiwan with *p53* mutations; 38% of the mutations were G:C→A:T transitions and 25% silent. The sites of *p53* mutations were mainly exons 5 and 8.

In contrast to *p53* mutation, no mutation was observed for *p16* in the peripheral blood samples of 60 patients, including 5 patients with *p53* mutations, even in the mutation hotspot exon 2 (Kamb et al. 1994; Nobori et al. 1994). We recognize that mutations of *p16* may have been observed if we had analyzed more samples. Previously, expression of P16 protein was observed in tumor tissues, but in our study we used peripheral blood samples. Furthermore, other exon mutations or inactivation mechanisms such as deletion or methylation could have inactivated *p16* (Lu et al. 2004; Zhao et al. 2003). We have recently observed *p16* exons 1 and 2 deletions using multi-PCR in peripheral blood of arsenicosis patients (*n* = 41) from blood samples obtained in 2004 (Bin et al. 2006). In noncarcinoma patients (*n* = 23), 8.7% exhibited *p16* deletions. In comparison, 38.9% of carcinoma patients (*n* = 18) showed *p16* deletions. This difference was statistically significant (*p* < 0.05).

### DNA synthesis, repair, and As carcinogenicity

Arsenic exposure has a profound impact on DNA synthesis. This is best illustrated by a 4-fold decrease in the DNA synthesis rate when the external and internal control groups were compared (Table 1). The unscheduled DNA synthesis showed a rather gradual increase as As exposure increased and symptoms worsened. It is plausible that As combines with the sulf-hydryl group of DNA polymerase and repairase, which inhibits the activity of DNA polymerase and repairase (Meng 1998).

Our study also found that As exposure has a profound negative impact on DNA repair, decreasing positive expression for XRCC1, MGMT*,* and hMSH2 mRNA with the increasing severity of skin lesion in arsenicosis patients (Table 3). The expression product of XRCC1 is essential in repairing DNA defects caused by ionizing radiation and alkylating agents. XRCC1 is involved in base excision and single-strand break repair (Whitehouse et al. 2001). Cells with XRCC1 deletion were found to be sensitive to ionization radiation and had a high SCE ratio (Zheng 1999). MGMT is a highly specific repair enzyme involved in repairing the base at the site of guanine O^6^ by transferring the alkylated base to the cysteine residue (Estellen et al. 2000). The natural expression of MGMT is a key in maintaining DNA stability of the DNA structure and repairing DNA alkylation damage. hMSH2 mRNA can repair a variety of DNA damage, including alkyl-base mismatches, insertions or deletions, and structural abnormalities, which lowers the mutation frequency and maintains stability of DNA structure.

Our study showed that long-term As exposure may be associated with chromosome and DNA damage, gene mutations, deletions, and alterations in DNA synthesis and repair. However, the mechanim(s) by which cellular and genetic damage leads to cancer remains unclear and further research is needed.

## Figures and Tables

**Table 1:** Chromosome and DNA damage [mean ± SD (*n*)] in 184 villagers from Jiaole township (internal control and exposed groups) compared with 53 villagers from Ma Jiatun township (external control).

	As concentration (mean ± SD)		Chromosome damage			DNA damage			
Group	Urinary (μg/L)	Hair (μg/g)	SCE	CA (%)	MN (‰)	DS (cpm)	DPC (cpm/μg DNA)	UDS (cpm)	DNA comet length (μm)
External control	45.6 ± 15.7 (53)	1.6 ± 1.2 (45)	3.0 ± 0.9 (23)	6.1 ± 2.3 (19)	1.5 ± 0.8 (25)	1,153 ± 710 (39)	943 ± 181 (40)	515 ± 196 (33)	8.6 ± 4.1 (41)
Internal control	76.3 ± 40.8^a^ (16)	5.4 ± 4.9^a^ (16)	3.8 ± 0.8^a^ (10)	10.8 ± 6.0 (13)	1.8 ± 1.1 (12)	254 ± 113^a^ (12)	1,229 ± 336^a^ (11)	611 ± 362 (12)	13.8 ± 8.8^b^ (19)
Mild	121.1 ± 110.3^a^,^d^ (45)	6.7 ± 6.1^a^ (46)	4.1 ± 1.3^a^ (16)	15.1 ± 6.8^a^ (43)	1.9 ± 1.3 (44)	315 ± 197^a^ (19)	1,611 ± 476^a^ (21)	687 ± 387^b^ (21)	19.5 ± 6.7^a^,^d^ (40)
Intermediate	140.2 ± 135.5^a^,^d^ (51)	7.5 ± 7.0^a^ (48)	4.6 ± 1.0^a^ (40)	15.6 ± 9.1^a^ (35)	1.8 ± 1.1 (49)	299.2 ± 255.2^a^ (31)	2,130 ± 776^a^,^c^,^f^ (25)	744 ± 507^b^ (29)	30.1 ± 12.6^a^,^c^ (54)
Severe	145.2 ± 128.5^a^,^d^ (55)	7.9 ± 7.8^a^ (62)	4.8 ± 1.1^a^,^d^ (56)	17.2 ± 12.4^a^ (59)	2.4 ± 1.4^a^,^d^,^f^,^g^ (62)	213 ± 132^a^ (39)	2,737 ± 1,017^a^,^c^,^a^,^a^ (40)	782 ± 440^b^ (38)	38.2 ± 14.4^a^,^c^ (62)

Abbreviations: CA, chromosome aberration; DPC, DNA–protein cross-link; DS, spontaneous DNA synthesis; MN, micronucleus; *n* = number of subjects in each group; SCE, sister chromatid exchange; UDS, unscheduled DNA synthesis.

a Significantly different compared with the external control group (*p* < 0.01 using ANOVA).

b Significantly different compared with the external control group (*p* < 0.05 using ANOVA).

c Significantly different compared to the internal control group (*p* < 0.01 using ANOVA).

d Significantly different compared with the internal control group (*p* < 0.05 using ANOVA).

e Significantly different compared with the mild group (*p* < 0.01 using ANOVA).

f Significantly different compared with mild group (*p* < 0.05 using ANOVA).

g Significantly different compared with the intermediate group (*p* < 0.05 using ANOVA).

**Table 2:** Expressions of P53 and P16 proteins and *p53* gene mutations in 70 villagers from Jiaole township.

	As concentration (mean ± SD)			P53		P16
Group	*n*	Urinary (μg/L)	Hair (μg/g)	Protein positive rate [positive/total (%)]	Gene mutation frequency [mutation/total (%)]	Protein positive rate [positive/total (%)]
Common pathological changes	22	134.0 ± 49.3	8.1 ± 6.5	3/18 (17)	0/22 (0)	15/19 (79)
Hyperkeratosis	22	146.1 ± 89.4	8.9 ± 4.8	7/21 (33)	0/16 (0)	15/22 (68)
Precarcinoma	12	146.2 ± 51.1	9.6 ± 5.3	8/11 (73)^a^,^b^	2/12 (17)^a^,^b^	4/11 (36)^a^
Carcinoma	18	169.8 ± 134.9	10.5 ± 9.4	16/18 (89)^c^,^d^	3/10^e^ (30)^a^,^b^	6/18 (33)^b^,^c^

*n* = number of subjects in each group.

a *p* < 0.05 compared with common pathological changes group using the chi-square test.

b *p* < 0.05 compared with hyperkeratosis group using the chi-square test.

c *p* < 0.01 compared with common pathological changes group using the chi-square test.

d *p* < 0.01 compared with hyperkeratosis group using the chi-square test.

e Two examples are silent mutations in three mutation samples.

**Table 3:** Comparison of the expression of DNA repair genes in 61 villagers with different skin lesion symptoms from Jiaole township, XinRen county, Guizhou province, China.

		As concentration (mean ± SD)		DNA repair genes [no. (%)]		
Group	*n*	Urinary (μg/L)	Hair (μg/g)	MGMT Positive/total	XRCC1 Mutation/total	hMSH2 Positive/total
Common pathological changes	22	144.7 ± 56.5	9.9 ± 5.0	16/22 (72.7)	17/22 (77.3)	16/22 (72.7)
Hyperkeratosis	16	156.0 ± 70.7	11.0 ± 5.0	11/16 (68.8)	10/16 (62.5)	12/16 (75.0)
Precarcinoma	12	164.9 ± 64.4	11.5 ± 5.4	5/12 (41.7)	5/12 (41.7)	8/12 (66.7)
Carcinoma	11	169.5 ± 75.3	12.0 ± 5.1	2/11 (18.2)^a^,^b^	3/11 (27.3)^a^	4/11 (36.4)

*n* = number of subjects in each group.

a *p* < 0.01 compared with common pathological changes group using the chi-square test.

b *p* < 0.05 compared with hyperkeratosis group using the chi-square test.

**Table 4:** *p53* gene codon mutation types.

Codon	Wild-type	Mutation type	Amino acids	Sample size (*n*)
143	GTG	GTA	Silent	2
146	TGG	CGG	Trp→Arg	1
151	CCC	TCC	Pro→Ser	2
